# Predictive Value of Frailty on Outcomes of Patients With Cirrhosis: Systematic Review and Meta-Analysis

**DOI:** 10.2196/60683

**Published:** 2025-02-27

**Authors:** Wen-Zhen Tang, Sheng-Rui Zhu, Shu-Tian Mo, Yuan-Xi Xie, Zheng-Ke-Ke Tan, Yan-Juan Teng, Kui Jia

**Affiliations:** 1 Department of Hepatobiliary Surgery, The First Affiliated Hospital of Guangxi Medical University, Nanning, Guangxi Zhuang Autonomous Region, China; 2 Department of Central Sterile Supply, The First Affiliated Hospital of Guangxi Medical University,, Nanning, Guangxi Zhuang Autonomous Region, China; 3 Nursing Department, The First Affiliated Hospital of Guangxi Medical University, Nanning, Guangxi Zhuang Autonomous Region, China; 4 Department of Gastrointestinal Surgery, The First Affiliated Hospital of Guangxi Medical University, 6 Shuangyong Road, Nanning, Guangxi Zhuang Autonomous Region, 530021, China, +86 0771-12580-6

**Keywords:** frailty, cirrhosis, diagnostic efficiency, survival, meta-analysis, prognostic factor, systematic review

## Abstract

**Background:**

Frailty is one of the most common symptoms in patients with cirrhosis. Many researchers have identified it as a prognostic factor for patients with cirrhosis. However, no quantitative meta-analysis has evaluated the prognostic value of frailty in patients with cirrhosis.

**Objective:**

This systematic review and meta-analysis aimed to assess the prognostic significance of frailty in patients with cirrhosis.

**Methods:**

The systematic review was conducted in accordance with PRISMA (Preferred Reporting Items for Systematic Reviews and Meta-Analyses) recommendations. We conducted a comprehensive search of the literature using databases such as PubMed, Cochrane Library, Embase, and Web of Science, as well as China National Knowledge Infrastructure, encompassing the period from inception to 22 December 2023. Data were extracted for frailty to predict adverse outcomes in patients with cirrhosis. RevMan (version 5.3) and R (version 4.2.2) were used to assess the extracted data.

**Results:**

A total of 26 studies with 9597 patients with cirrhosis were included. Compared with patients having low or no frailty, the frail group had a higher mortality rate (relative ratio, RR=2.07, 95% CI 1.82‐2.34, *P*<.001), higher readmission rate (RR=1.50, 95% CI 1.22‐1.84, *P*<.001), and lower quality of life (RR=5.78, 95% CI 2.25‐14.82, *P*<.001). The summary receiver operator characteristic (SROC) curve of frailty for mortality in patients with cirrhosis showed that the false positive rate (FPR) was 0.25 (95% CI 0.17-0.34), diagnostic odds ratio (DOR) was 4.17 (95% CI 2.93-5.93), sensitivity was 0.54 (95% CI 0.39-0.69), and specificity was 0.73 (95% CI 0.64-0.81). The SROC curve of readmission showed that the FPR, DOR, sensitivity, and specificity were 0.39 (95% CI 0.17-0.66), 1.38 (95% CI 0.64-2.93), 0.46 (95% CI 0.28-0.64), and 0.60 (95% CI 0.28-0.85), respectively.

**Conclusions:**

This meta-analysis demonstrated that frailty is a reliable prognostic predictor of outcomes in patients with cirrhosis. To enhance the prognosis of patients with cirrhosis, more studies on frailty screening are required.

## Introduction

### Background

Cirrhosis is a prevalent illness worldwide and is linked with substantial morbidity and mortality [1,2]. According to a recent report by Smith et al [3], cirrhosis has resulted in the deaths of over 40,000 individuals in America, ranking it as the 12th most prevalent cause of death. Furthermore, it is the third most prevalent cause of mortality among individuals aged 45‐64 years [4]. The global annual mortality from chronic liver disease is estimated to be approximately 2 million. Amongst them, cirrhosis is associated with the advancement of chronic inflammatory diseases and accounts for approximately 45% of all-cause mortality worldwide [5].

Cirrhosis often causes physiological decline, making patients more vulnerable to frailty, which further compromises their overall health [6]. Frailty is a clinical syndrome defined by increased susceptibility and impaired antistress response due to the cumulative functional loss of numerous physiological systems [7]. Furthermore, frailty is characterized by a deterioration across three primary domains: physical health, mental health, and social function [8]. Previously, frailty was considered primarily a geriatric condition linked with aging, although its link to chronic diseases is now extensively understood [9-11].

At present, evidence is increasing that frailty is a risk factor for increased mortality and complications, longer hospital stays, risk of falls, and other issues in patients with cirrhosis [12-14]. However, the assessment tools used in prior research on the unfavorable consequences of patients with cirrhosis vary, and more investigation is required to determine the prognostic significance of frailty in these individuals [12,15]. The prognostic significance of frailty in patients with cirrhosis is inconsistent among different studies and requires immediate clarification [16,17]. For example, several researchers have demonstrated that frailty serves as a prognostic indicator for mortality in individuals with cirrhosis [17,18], but conflicting findings have been reported in another investigation [16]. In addition, one study explored the relationship between frailty and outcome in patients with cirrhosis, but it only demonstrated that frailty can predict liver transplant-free survival; the risk of other complications remains unknown [19]. There are still limited systematic reviews and meta-analyses on the prognostic value of frailty in patients with cirrhosis.

### Objective

Therefore, to determine the association between frailty and the prognosis of patients with cirrhosis, we did a comprehensive review and meta-analysis on the predictive value of frailty in the prognosis of patients with cirrhosis. This information can help develop targeted management measures for patients with cirrhosis and promote their well-being.

## Methods

### Protocol Registration

The study protocol was officially registered on the PROSPERO website (No. CRD42024497698).

### Search Strategy

A comprehensive search was conducted in PubMed, Cochrane Library, Embase, and Web of Science, as well as the China Knowledge Resource Integrated Database, to identify potential articles describing frailty and cirrhosis from inception until 22 December 2023. The search method was conducted with a combination of keywords and Mesh terms: (“frailty” or “frail”) and (“liver cirrhosis,” “hepatic cirrhosis,” “liver fibrosis,” “cirrhosis,” or “cirrhotic”). In addition, we conducted a thorough manual examination of the reference lists of relevant primary and secondary research papers. The search strategy is demonstrated in Table S1 in Multimedia Appendix 1.

### Study Selection

The following were the inclusion criteria: (1) cohort studies revealed details about the frailty among patients with cirrhosis; (2) patients (18 years or above) diagnosed with cirrhosis; (3) frailty was assessed using a standardized and reliable instrument, such as the fried frailty score (FFS) and liver frailty index (LFI); (4) at least one clinical outcome between cirrhosis with and without frailty was reported during follow-up; and (5) publications were in English or Chinese.

The following were the exclusion criteria: (1) reviews, non-cohort studies, letters, and conference abstracts; (2) studies that did not provide complete data; (3) duplicated papers; (4) studies not reporting the prevalence of frailty or the predictive value of frailty on the outcomes of patients with cirrhosis; and (5) patients included other diseases rather than cirrhosis

### Data Extraction

Author and publication year, country, study design, study population, sample size, age, follow-up (months), frailty instruments, and prevalence of frailty were extracted by two researchers working independently. For each study, the relative ratio (RR) and 95% CI were extracted, both with and without adjustment for confounding variables. Furthermore, from the studies that were incorporated, the true positive (TP), false positive (FP), true negative (TN), and false negative (FN) values were extracted.

### Quality Assessment

Two authors independently assessed the cohort studies using the Newcastle–Ottawa Scale (NOS) instrument [20]. It contained three components, including the study group selection, group comparability, and ascertainment of outcomes. The instrument had a maximum score of 9, with a score higher than 5 indicating high-quality literature [21]. If discrepancies existed, the corresponding author assisted in reaching a consensus.

### Statistical Analysis

The meta-analysis was conducted using Review Manager (version 5.3) and R software (version 4.2.2; R Foundation for Statistical Computing).

For the purpose of estimating the effect size, the RR and 95% CI were used. The *χ*^2^ test was used to investigate heterogeneity among the outcomes of each study, and the *I*^2^ test was utilized to quantify the level of heterogeneity. The funnel plot was used to evaluate the publication bias when the number of publications reached or exceeded 10. When there were less than 10 publications, the Egger test was used to evaluate literature bias. Further investigation was conducted using the trim-and-fill method if the published literature was biased. Statistical significance was considered to be indicated by *P* values less than .05.

The average sensitivity, specificity, false-positive rate (FPR), positive likelihood ratio (LR+), negative likelihood ratio (LR-), and diagnostic odds ratio (DOR) of the included studies were computed using the random-effect model. Furthermore, the accuracy of the test and the consistency of the outcomes of the included studies were determined using the summary receiver operating characteristic (SROC) curve.

### Ethical Considerations

All analyses were conducted using previously published studies, and therefore, ethics approval and patient consent were unnecessary. This review does not include human subject information, primary data collection, or any form of experimentation involving individuals.

## Results

### Selected Studies

**Figure 1.**
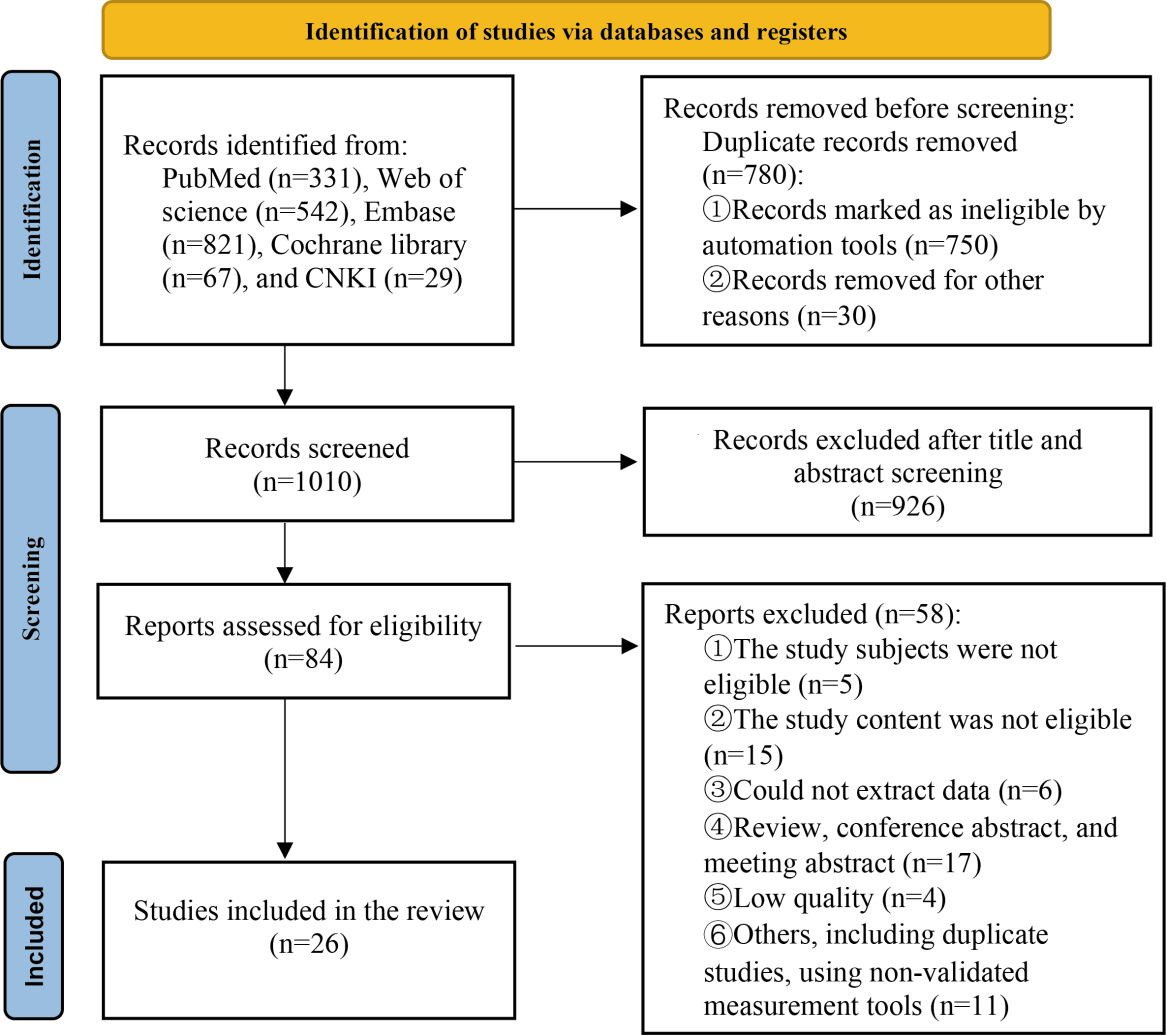
Flowchart showing the database search.

Following the identification of 1790 articles during the preliminary search, 780 duplicate articles were eliminated. By reviewing titles and abstracts, an additional 926 sources were eliminated from consideration primarily owing to their lack of relevance to the meta-analysis. The remaining 84 studies were read completely. A total of 58 were deemed ineligible for the reasons outlined in Figure 1. Finally, 26 studies were considered in this analysis. The search flow of this study is presented in Figure 1; the PRISMA (Preferred Reporting Items for Systematic Reviews and Meta-Analyses) checklist has been shown as Checklist 1.

### Characteristics of the Included Studies

As shown in Table 1, the meta-analysis comprised 26 cohort studies with 9597 patients with cirrhosis [12,13,15,18,22-43]. Among the 26 articles, 3 were retrospective studies [13,18,41] and 23 were prospective studies [12,15,22-40,42,43]. They were published between 2013 and 2023, and performed in the USA [13,15,18,27,30-32,35,40,41], Canada [39,43], Chile [38], China [22-25,33], Egypt [12], Germany [26,28,29], India [34], the Netherlands [42], Slovakia [37], and Thailand [36]. Considering the included patients, 15 were patients with cirrhosis [12,22-26,29,32-34,36-40], 8 were patients with cirrhosis waiting for liver transplants, 3 were patients who received liver transplants [13,35,41], and the remaining 1 patient had cirrhosis after surgery [18]. Moreover, the number of patients included was between 88 and 9597. A variety of tools were used to diagnose frailty, including the short physical performance battery (SPPB) [12,39], Carolina frailty index (CFI) [22,25], fried frailty score (FFS) [15,28,37], liver frailty index (LFI) [23,26,27,30-32,34-37,43], clinical frailty scale (CFS) [29,39], gait speed test (GST) [13,32,40], and others [18,24,33,38,41]. The mean follow-up durations varied between 1 and 60 months. The prevalence of frailty in patients with cirrhosis ranged from 10.50% to 75.40%. The NOS scores of the studies included in the study varied between 6 and 8, indicating a high level of quality in these studies. Table S2 in Multimedia Appendix 1 displays the evaluation criteria for literature quality.

**Table 1.** Basic characteristics of the included studies (n=26).

Author, year (citation)	Country	Study design	Study population	Sample size	Age, years	Follow-up (months)	Frailty tools	Prevalence of frailty	Quality assessment score
Behiry, 2018 [12]	Egypt	Prospective	Patients with cirrhosis	145	mean (SD), 60 (7)	3	SPPB^a^	-	6
Deng, 2020 [22]	China	Prospective	Patients with cirrhosis	158	mean (range), 64 (57-70)	24	CFI^b^	14.56%	8
Dunn, 2016 [15]	USA	Prospective	Patients with cirrhosis waiting for liver transplant	373	mean (SD), 56.7 (10.1)	3	FFS^c^	-	7
Guo, 2022 [23]	China	Prospective	Patients with cirrhosis	221	mean (range), 63 (57–68)	24	LFI^d^	14.50%	7
Hui, 2022 [24]	China	Prospective	Patients with cirrhosis	227	mean (SD), 61.7 (9.9)	48	FI^e^	-	7
Hui, 2021 [25]	China	Prospective	Patients with cirrhosis	105	mean (SD), 61.6 (9.5)	-	CFI^f^		6
Kaps, 2022 [26]	Germany	Prospective	Patients with cirrhosis	88	mean (range), 60 (51-67)	1	LFI	51%	8
Kardashian, 2021 [27]	USA	Prospective	Patients with cirrhosis waiting for liver transplants	1405	mean (range), 57 (49-63)	12	LFI	-	7
Klein, 2021 [28]	Germany	Prospective	Patients with cirrhosis waiting for liver transplants	114	mean (range), 53 (42-60)	48	FFS	75.40%	6
Kremer, 2020 [29]	Germany	Prospective	Patients with cirrhosis	200	mean (range), 60 (52‐66)	24	CFS	10.50%	7
Lai, 2018 [30]	USA	Prospective	Patients with cirrhosis waiting for liver transplants	529	mean (range), 58 (50‐63)	24	LFI	-	6
Lai, 2022 [31]	USA	Prospective	Patients with cirrhosis waiting for liver transplants	1166	mean (range), 60 (53‐64)	60	LFI	94%	7
Lin, 2022 [32]	USA	Prospective	Patients with cirrhosis	116	mean (SD), 56 (11)	48	LFI, 6MWT^g^, GST^h^	25%	6
Luo, 2023 [33]	China	Prospective	Patients with cirrhosis	285	mean (SD), 59.1 (12.3)	36	FFP^i^	37.20%	6
Mahmud 2021 [18]	USA	Retrospective	Patients with cirrhosis undergoing surgery	804	-	36	HFRS^j^	48.50%	6
Nathiya 2023 [34]	India	Prospective	Patients with cirrhosis	156	mean (SD), 47.42 (13.47)	-	LFI	44.92%	7
Salim 2020 [13]	USA	Retrospective	Patients with cirrhosis who received liver transplants	107	mean (SD), 58 (11)	1	GST, CST^k^	37.80%	7
Serper 2021 [35]	USA	Prospective	Patients with cirrhosis who received liver transplants	211	mean (SD), 57 (12)	8	LFI	59%	8
Siramolpiwat 2021 [36]	Thailand	Prospective	Patients with cirrhosis	152	mean (SD), 62.5 (9.3)	18	LFI	24.30%	8
Skladany 2021 [37]	Slovakia	Prospective	Patients with cirrhosis	168	mean (SD), 57.9 (14.3)	6	LFI, CFS, FFS,SPPB	-	7
Soto 2021 [38]	Chile	Prospective	Patients with cirrhosis	126	mean (SD), 64 (8.3)	48	FFP	65.10%	7
Tandon 2016 [39]	Canada	Prospective	Patients with cirrhosis	300	mean (SD), 57.4 (9.3)	6	CFS, FFC^l^, SPPB	18%	8
Tapper 2015 [41]	USA	Retrospective	Patients with cirrhosis who received liver transplants	734	mean (SD), 57.3 (11.5)	3	ADL^m^, BS^n^, MFS^o^	-	6
Tapper 2019 [40]	USA	Prospective	Patients with cirrhosis	300	mean (range), 60 (52‐66)	-	GST and CS	-	8
van Vugt 2017 [42]	Netherlands	Prospective	Patients with cirrhosis waiting for liver transplants	585	mean (range), 56 (48‐62)	3	The MELD-Sarcopenia	43.40%	7
Wang2021 [43]	Canada	Prospective	Patients with cirrhosis waiting for liver transplants	822	mean (SD), 55.2 (9.9)	-	LFI	24.40%	6

a SPPB: short physical performance battery.

b CFI: Carolina frailty index.

c FFS: Fried frailty score.

d LFI: liver frailty index.

e FI: frailty index.

f CFS: clinical frailty scale.

g 6MWT: 6-minute walk test.

h GST: gait speed test.

i FFP: fried frailty phenotype.

j HFRS: hospital frailty risk score.

k CST: chair stands test.

l FFC: fried frailty criteria.

m ADL: activities of daily living.

n BS: Braden scale.

o MFS: Morse fall scale.

### Meta-Analysis Results

#### Mortality

**Figure 2.**
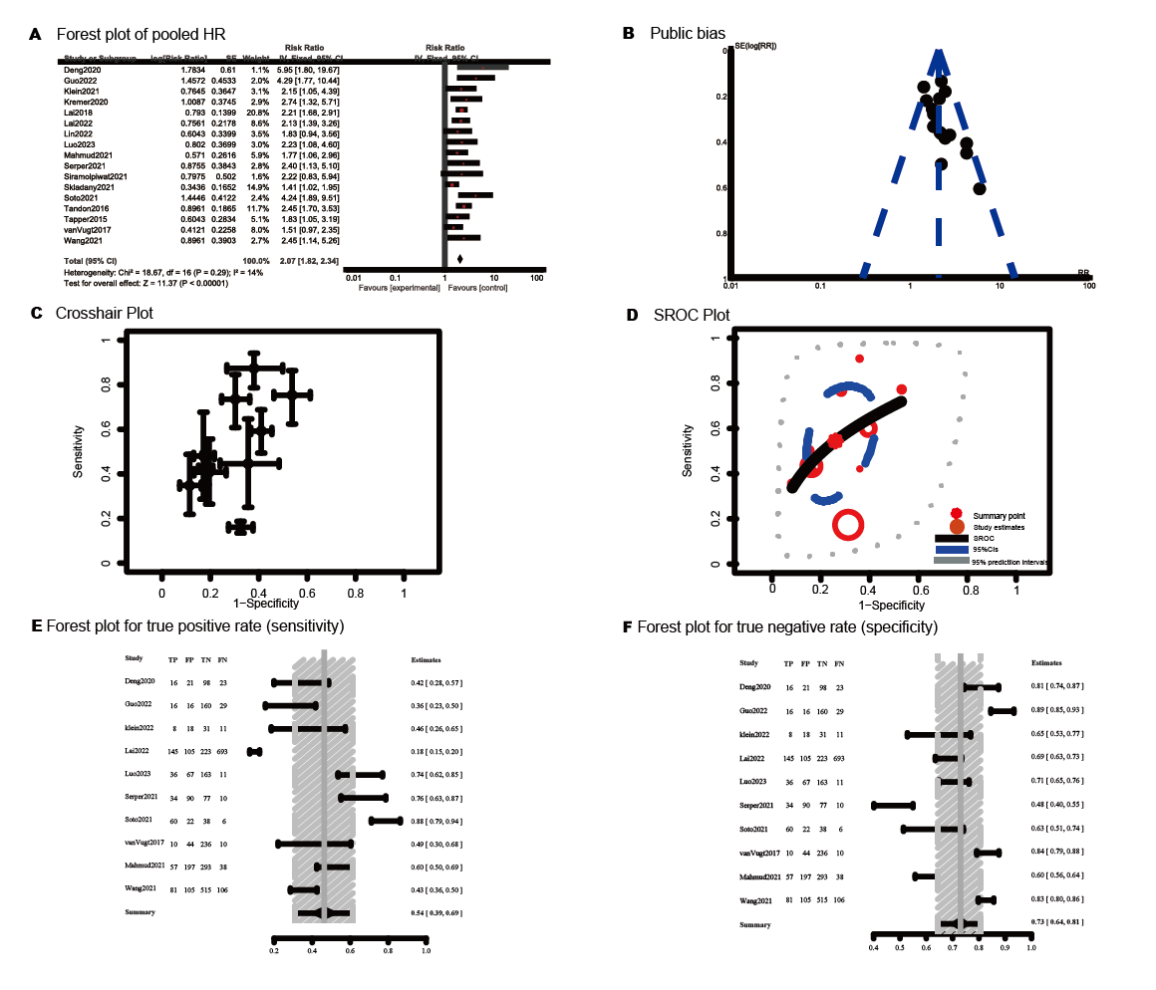
Pooled diagnostic parameters of frailty for the mortality of patients with cirrhosis [18,22,23,28-33,35,36,38,39,42,43]. (A) Forest plot of the relationship between frailty and mortality in patients with cirrhosis; (B) Funnel plot of the included studies; (C) Crosshair plots of each study of frailty in the prediction for mortality in patients with cirrhosis; (D) Summary receiver operating characteristic (SROC) plots of frailty in the prediction for mortality in patients with cirrhosis; (E) Forest plots of the sensitivity of each individual study; (F) Forest plots of the specificity of each individual study.

Seventeen studies reported the association between frailty and mortality in patients with cirrhosis [18,22,23,28-33,35,36,38,39,42,43]. Pooled results using a fixed-effect model indicated a significant correlation between frailty and mortality (RR 2.07, 95% CI 1.82‐2.34, *P*<.001, I^2^=14%; Figure 2A). A visual examination revealed that the funnel plot was symmetrical, suggesting a low probability of publishing bias (Figure 2B). The crosshair plots displayed the sensitivity, FPR, and 95% CI of the included studies (Figure 2C) [22,23,29,31,33,35,38,39,42,43]. The SROC curve with 95% CI and 95% prediction intervals of frailty in cirrhosis is shown in Figure 2D. The FPR was 0.25 (95% CI 0.17-0.34), and the DOR was 4.17 (95% CI 2.93-5.93). The summary LR+ was 2.35 (95% CI 1.86-2.96) and the LR- was 0.56 (95% CI 0.45-0.71). As shown in Figure 2E, frailty had a summary sensitivity of 0.54 (95% CI 0.39-0.69). The combined specificity of all studies was 0.73 (95% CI 0.64-0.81; Figure 2F).

Additional subgroup analyses revealed that the frailty-assessment methods, sample size, and study population may not have substantially affected the results (Table 2).

**Table 2.** Subgroup analyses for the association between frailty and mortality in patients with cirrhosis.

Groups		No of studies	*I* ^*2*^	RR	95% CI	*P* value
Assessment tool						
	CFI^a^	3	0	2.51	1.81-3.48	<.001
	LFI^b^	7	0	2.25	1.86-2.74	<.001
	SPPB^c^	2	92	1.29	1.20-1.38	<.001
	FFS^d^	2	0	1.71	1.41-2.08	<.001
	CFS^e^	3	62	2.04	1.44-2.88	<.001
Sample						
	≥200	11	0	2.15	1.86-2.49	<.001
	<200	6	49	1.77	1.52-2.06	<.001
Study population						
	Patients with cirrhosis	9	48	2.13	1.76-2.58	<.001
	Patients with cirrhosis waiting for liver transplants	5	0	2.05	1.70-2.48	<.001
	Patients with cirrhosis who received liver transplants	2	0	2.01	1.29-3.15	.002

a CFI: Carolina frailty index.

b LFI: liver frailty index.

c SPPB: short physical performance battery.

d FFS: fried frailty score.

e CFS: clinical frailty scale.

#### Readmission

**Figure 3.**
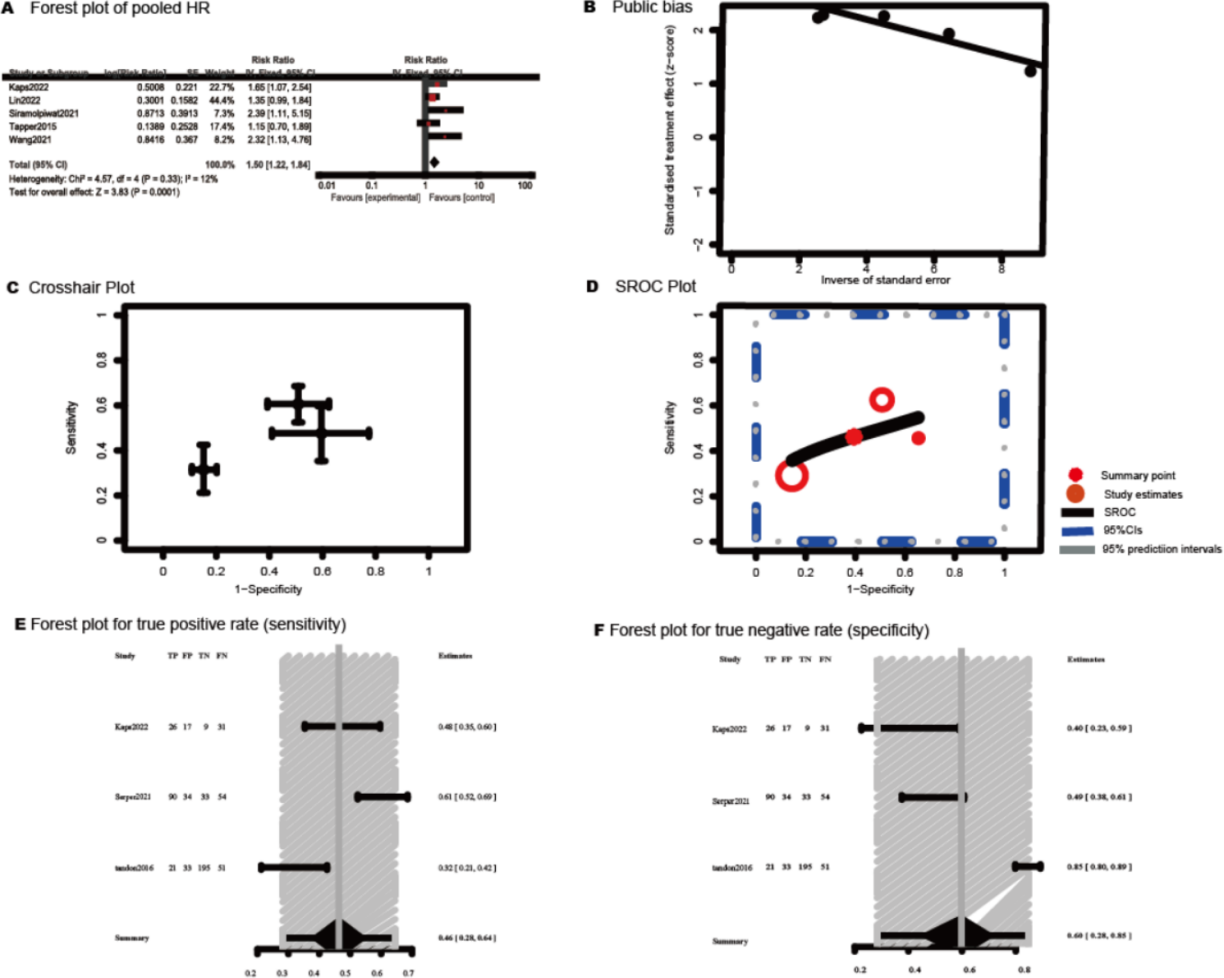
Pooled diagnostic parameters of frailty for readmission in patients with cirrhosis [26,32,36,41,43]. (A) Forest plot of the relationship between frailty and readmission in patients with cirrhosis; (B) Egger test plot of the included studies; (C) Trim-and-fill plot of the included studies; (D) Crosshair plots of each study of frailty in prediction readmission in patients with cirrhosis; (E) Summary receiver operating characteristic (SROC) plots of frailty in prediction for readmission in patients with cirrhosis; (F) Forest plots of the sensitivity of each individual study; (G) Forest plots of the specificity of each individual study.

Figure 3A displays the inclusion of 5 studies in the pooled analysis investigating the relationship between frailty and readmission in patients with cirrhosis [26,32,36,41,43]. The pooled results with a fixed-effect model (I^2^=12%, *P*=.33) indicated that patients with cirrhosis with frailty had an increased risk of readmission (RR 1.50, 95% CI 1.22‐1.84, *P*<.001). The 5 included publications had a significant bias, according to the Egger test results (*t*=13.64, *P*<.001, Figure 3B). Accordingly, we further conducted trim-and-fill analysis, and results showed that the effect size was 1.31 (95% CI 1.01-1.70, *P*=.042, Figure 3C). This result showed that substantial relationships were unchanged when potential publication bias was taken into account.

Three studies provided descriptions of the TP, FP, FN, and TN [26,35,39]. Accordingly, we conducted a diagnostic test accuracy for them. Crosshair plots were drawn to display the sensitivity (FPR and 95% CI of the 3 studies, as shown in Figure 3D). The SROC curve is shown in Figure 3E, with the FPR of 0.39 (95% CI 0.17-0.66) and the DOR of 1.375 (95% CI 0.64-2.93). The summary LR+ was 1.200 (95% CI 0.74-1.94), and the LR- was 0.873 (95% CI 0.66-1.16). The combined sensitivity and specificity of all included studies were 0.46 (95% CI 0.28-0.64) and 0.60 (95% CI 0.28-0.85), as shown in Figure 3F and G.

#### Quality of Life

**Figure 4.**
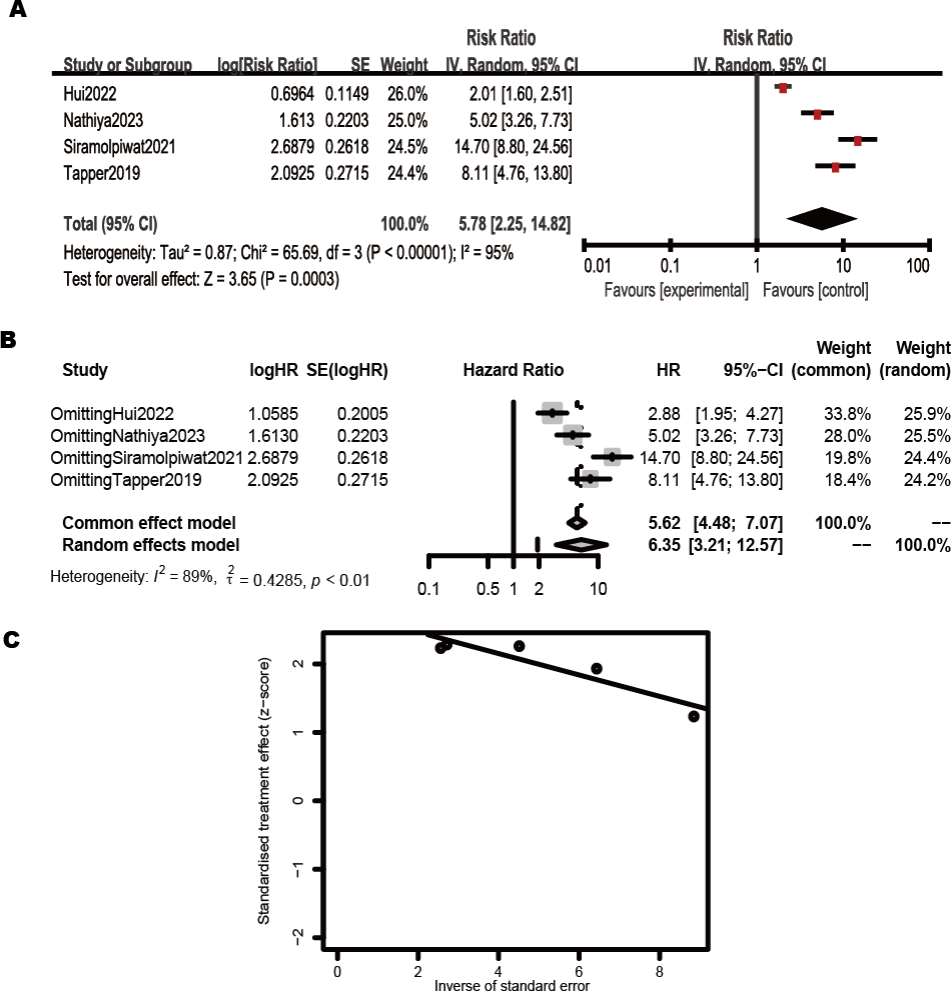
Pooled diagnostic parameters of frailty for decreasing the quality of life (QoL) in patients with cirrhosis [24,34,36,40]. (A) Forest plot of relationship between frailty and the QoL in patients with cirrhosis; (B) Sensitivity analysis plot of the included studies; (C) Egger test plot of the included studies.

Four studies examined the correlation between frailty and the quality of life (QoL) in patients with cirrhosis [24,34,36,40]. The pooled results of 4 studies with random-effect models indicated that frailty was significantly associated with decreased QoL in patients with cirrhosis (RR 5.78, 95% CI 2.25-14.82, *P*<.001, I^2^=95%, Figure 4A). Sensitivity analysis, which eliminated studies one by one, revealed that the results were consistent (RR 6.35, 95% CI 3.21‐12.57, *P*<.01, Figure 4B). The results of the Egger test indicated no obvious bias in the included 4 articles (*t*=3.08, *P*=.091, Figure 4C).

#### Descriptive Analysis

Descriptive analyses were carried out because the following results were given in only 2 or fewer studies. Frailty was associated with non-home charge [31,35], prolonged hospital days [15,31], decompensation [33,36], sleep disturbance [25], prolonged posttransplant intensive care unit days [31], aspiration [13], and extended intubation days [13].

## Discussion

### Principal Results and Comparison With Previous Works

In this meta-analysis, our main findings demonstrated that patients with cirrhosis with frailty had a significantly higher risk of mortality, higher readmission, and lower QoL than those without frailty. According to our evaluation of the literature, the current study provided the most thorough evidence that frailty was a predictor of unfavorable clinical outcomes in patients with cirrhosis.

In this study, the prevalence of frailty in patients with cirrhosis ranged from 10.50% to 75.40%, depending on the specific techniques used to diagnose frailty, the characteristics of the study participants, and the operational definitions used. For example, the study conducted by Lai used the LFI to evaluate frailty in patients with cirrhosis and found that it has a prevalence rate of 94% [31], while another study used the CFI to assess frailty, and results show that the prevalence is only 14.56% [22]. Sensitivity and specificity are influenced by the assessment instrument and the chosen threshold [44]. When selecting frailty-assessment tools for patients with cirrhosis, understanding the content of existing frailty-assessment tools and the suitable population is critical to choosing the most appropriate frailty-screening tool. Furthermore, the instrument most frequently reported in this research was the LFI, a specialized tool designed to evaluate the frailty of liver disease. Other assessment tools included the SPPB, CFI, FFS, CFS, and 6-minute walk test (6MWT). Each of these tool tests is a performance-based measure that necessitates active patient participation, restricting their applicability in patients with severe or acute decompensation [45]. At present, researchers still debate about which evaluation tool for cirrhosis faltering is the standard, and a significant number of relevant studies are required to confirm it in the future.

This meta-analysis demonstrated that frailty was more likely linked to poor survival in patients with cirrhosis, consistent with previous studies [46]. Subgroup analysis further revealed that frailty-assessment tools, sample size, and study subject characteristics may not significantly influence the association between frailty and mortality in patients with cirrhosis. Previous research has shown that patients with cirrhosis with frailty had a considerably worse prognosis than those without frailty, showing that frailty is a poor prognostic factor for chronic liver disease [47,48]. The mechanisms of frailty leading to poor prognosis in patients with cirrhosis include the upregulation of the inflammatory response, impaired immune function, low testosterone levels, intestinal flora disorder, decreased intestinal barrier function, and potential neuromuscular weakness [49-51]. For example, testosterone has been proven to be associated with frailty [52,53]. It is necessary to maintain physiological homeostasis, and its deficiency may decrease muscle strength, increasing the risk of falls, disability, and complications from acute illnesses [54]. In addition, the mechanisms involved in the relationship between frailty and unfavorable outcomes may increase susceptibility to complications, such as inflammatory insults [55], infection [56], and hepatic encephalopathy [13,57] in patients with cirrhosis, thereby affecting the survival of patients. However, the mechanism of action between frailty and cirrhosis prognosis is bidirectional, meaning that liver decompensation reimbursement increases the risk of frailty development [45]. Additionally, this study demonstrated that the probability of compensation in frail patients with cirrhosis was 2.55 times greater than that in non-frail patients. Specifically, only a limited number of studies have assessed the association between frailty and decompensation in patients with cirrhosis, and further investigation is necessary to delve into this association in the future.

This study’s findings suggested that patients with cirrhosis with frailty were more prone to have the risk of readmission than non-frail patients, as confirmed in other populations, including cancer patients [58,59], patients with chronic disease [60,61], and surgical patients [62,63]. A recent study conducted by Witt et al to evaluate the predictive value of frailty in 80 patients with chronic obstructive pulmonary disease has demonstrated that they have a higher risk of readmission than patients without frailty (OR 19.31, 95% CI 1.07‐349.03) [64]. This result may be explained by reduced physiological reserve, malnutrition, and impaired immune function in debilitated patients with cirrhosis [45,65]. For example, frail patients with reduced physiological reserve are more prone to complications and increased risk of infection when encountering stressors, which in turn increases the likelihood of patient readmission [66].

Our study provided some evidence of a correlation between frailty and diminished QoL among patients with cirrhosis. Frailty in patients with chronic liver disease is a multidimensional syndrome [49]. Frailty is characterized by the gradual decline of various physiological systems, such as the cardiovascular and musculoskeletal systems, which affects the patient’s daily activities and self-care ability and may increase the risk of death and hospitalization, thereby decreasing the QoL [34,49]. Two recent systematic reviews have shown a clear relationship between frailty, anxiety, and depression, which showed that frailty may lead to lower QoL levels by causing negative psychological effects [67,68]. Although this study found a significant decline in the QoL of patients with frailty due to the high heterogeneity, future larger and well-designed studies are needed to explore the relationship between frailty and QoL in patients with cirrhosis.

### Recommendations for Future Practice

An essential aspect of this study is to make health care professionals aware of the importance of frailty as one of the risk predictors of cirrhosis prognosis. Standardized assessment tools, such as the CFI and LFI, can identify the patients’ frailty status as early as possible to provide timely intervention. In addition, clinical staff can include frailty in the prognosis management plan of patients. In the meantime, personalized management measures can be formulated for patients with cirrhosis with different degrees of frailty, which can significantly improve the prognosis of patients. In addition, health care professionals should strengthen the positive management and follow-up of patients with cirrhosis, especially those with a higher degree of frailty, determine the abnormal situation of patients with cirrhosis in time, adjust the management plan, and ultimately improve the prognosis of patients.

### Implications for Future Research

This systematic review has important implications for future studies. If frailty is regarded as an important part of the routine assessment of patients with cirrhosis, researchers need to further compare the prognostic value of different frailty indicators in patients with cirrhosis and reach a consensus on the assessment tools for frailty in patients with cirrhosis. In addition, we suggest that future studies should target the protective factors of frailty to inform preventive strategies for patients with cirrhosis. In addition, researchers and policy makers should develop comprehensive treatment approaches and strengthen interdisciplinary collaboration to provide better management strategies for frailty in patients with cirrhosis.

### Strength and Limitations

This was the first systematic review to examine the predictive value of unfavorable outcomes of frailty in patients with cirrhosis. We established that frailty was a good prognostic factor of the clinical outcomes in patients with cirrhosis, including mortality, readmission, and decreased QoL. A systematic review by Bowers et al revealed that several frailty assessments can reliably assess mortality in patients with cirrhosis who are ineligible for transplantation [69]. Future investigations should explore the trajectory of frailty change and its effect on outcomes across time. We aimed to perform a meta-analysis on the longitudinal patterns of frailty and their association with outcome measures.

This study had a few limitations. First, we included only literature reported in Chinese and English, which may have left out some relevant studies in other languages. Second, our study had an inadequate sample size, necessitating higher sample sizes in future research to evaluate the correlation between frailty and outcome in patients with cirrhosis. Furthermore, variations existed across the studies in the tools used to evaluate frailty, which may have influenced the outcomes.

### Conclusion

The results of this meta-analysis demonstrate a significant correlation between frailty and an unfavorable clinical outcome in patients with cirrhosis, specifically in terms of mortality and readmission. Future research should be conducted to further explore the most effective screening tools for diagnosing frailty in patients with cirrhosis, as well as whether frailty-related interventions were connected with better clinical outcomes in patients with cirrhosis.

## Supplementary material

10.2196/60683 Multimedia Appendix 1 The search strategy and quality assessment of the included studies.

10.2196/60683 Checklist 1 PRISMA checklist.

## Abbreviations

6MWT: 6-minute walk test
CFI: Carolina frailty index
CFS: clinical frailty scale
DOR: diagnostic odds ratio
FFS: fried frailty score
FN: false negative
FP: false positive
FPR: false-positive rate
GST: gait speed test
LFI: liver frailty index
LR: likelihood ratio
NOS: Newcastle-Ottawa Scale
PRISMA: Preferred Reporting Items for Systematic Reviews and Meta-Analyses
QoL: quality of life
RR: relative ratio
SPPB: short physical performance battery
SROC: summary receiver operating characteristic
TN: true negative
TP: true positive
