# Supplementary material for: Predictive Value of Frailty on Outcomes of Patients With Cirrhosis: Systematic Review and Meta-Analysis
Source: JMIR Med Inform. 2025 Feb 27;13:e60683. doi: 10.2196/60683 (PMC11912948; doi:10.2196/60683)
Supplement: Multimedia Appendix 1 [file medinform-v13-e60683-s001.pdf]

Table S1 Search strategy

**PubMed**
("frailty" OR "frail") AND ("Liver Cirrhosis" OR "Hepatic Cirrhosis" OR "Liver Fibrosis" OR "Cirrhosis" OR "cirrhotic"")
("frailty"[MeSH Terms] OR "frailty"[All Fields] OR "frailties"[All Fields] OR ("frail"[All Fields] OR "frails"[All Fields] OR "frailty"[MeSH Terms] OR "frailty"[All Fields] OR "frailness"[All Fields])) AND ((((("liver cirrhosis"[MeSH Terms] OR ("liver"[All Fields] AND "cirrhosis"[All Fields]) OR "liver cirrhosis"[All Fields]) AND "OR"[All Fields]) AND ("liver cirrhosis"[MeSH Terms] OR ("liver"[All Fields] AND "cirrhosis"[All Fields]) OR "liver cirrhosis"[All Fields] OR ("hepatic"[All Fields] AND "cirrhosis"[All Fields]) OR "hepatic cirrhosis"[All Fields])) AND "OR"[All Fields]) AND ("liver cirrhosis"[MeSH Terms] OR ("liver"[All Fields] AND "cirrhosis"[All Fields]) OR "liver cirrhosis"[All Fields] OR ("liver"[All Fields] AND "fibrosis"[All Fields]) OR "liver fibrosis"[All Fields]))
**Web of science**
#1 Search TS = ("Liver Cirrhosis" OR "Hepatic Cirrhosis" OR "Liver Fibrosis" OR "Cirrhosis" OR "cirrhotic")
#2 Search TS=("Frailty" OR "Frail") (Search within all fields of #1)
#3 #1 AND #2
**Embase**
#1 'liver cirrhosis'/exp OR 'liver cirrhosis' OR 'hepatic cirrhosis'/exp OR 'hepatic cirrhosis' OR 'liver fibrosis'/exp OR 'liver fibrosis' OR 'cirrhosis'/exp OR 'cirrhosis'
#2 'frailty'/exp OR frailty OR frail
#3: #1 AND #2
**Cochrane library 67**
#1 Search Title Abstract Keyword: frailty OR frail
#2 Search Title Abstract Keyword: Liver Cirrhosis OR Hepatic Cirrhosis OR Liver

Fibrosis OR Cirrhosis OR cirrhotic	
#3	Search #1 AND #2

Table S2 Quality assessment of the included studies

Study	Representativeness of the exposed cohort	Selection of the non-exposed cohort	Ascertainment of exposure	Demonstration that outcome of interest was no present as start of study	Comparability of cohort on the basis of the design or analysis	Assessment of outcome	Was follow-up long enough for outcomes to occur	Adequacy of follow up of cohorts	total quality score
Behiry2018	1	1	1	1	0	1	1	0	6
Deng2020	1	1	1	1	1	1	1	1	8
Dunn2016	1	1	1	1	1	1	1	0	7
Guo2022	1	1	1	1	0	1	1	1	7
Hui2022	1	1	1	1	1	1	1	0	7
Hui2021	1	1	1	1	0	1	1	0	6
Kaps2022	1	1	1	1	1	1	1	1	8
Kardashian2021	1	1	1	1	1	1	1	0	7
Klein2021	1	1	1	1	0	1	1	0	6
Kremer2020	1	1	1	1	1	1	1	0	7
Lai2018	1	1	1	1	0	1	1	0	6
Lai2022	1	1	1	1	1	1	1	0	7
Lin2022	1	1	1	1	0	1	1	0	6
Luo2023	1	1	1	1	0	1	1	1	7
Mahmud2021	1	1	1	1	0	1	1	0	6
Nathiya2023	1	1	1	1	0	1	1	1	7

Salim2020	1	1	1	1	1	1	1	0	7
Serper2021	1	1	1	1	1	1	1	1	8
Siramolpiwat2021	1	1	1	1	1	1	1	1	8
Skladany2021	1	1	1	1	0	1	1	1	7
Soto2021	1	1	1	1	0	1	1	1	7
Tandon2016	1	1	1	1	1	1	1	1	8
Tapper2015	1	1	1	1	1	0	1	0	6
Tapper2019	1	1	1	1	1	1	1	1	8
vanVugt2017	1	1	1	1	0	1	1	1	7
Wang2021	1	1	1	1	1	0	1	0	6
